# Antimicrobial Activities of Lipopeptides and Polyketides of *Bacillus velezensis* for Agricultural Applications

**DOI:** 10.3390/molecules25214973

**Published:** 2020-10-27

**Authors:** Muhammad Fazle Rabbee, Kwang-Hyun Baek

**Affiliations:** Department of Biotechnology, Yeungnam University, Gyeongsan, Gyeongbuk 38541, Korea; rabbi.biotech@gmail.com

**Keywords:** *Bacillus velezensis*, antimicrobial activity, lipopeptides, polyketides

## Abstract

Since the discovery of penicillin, bacteria are known to be major sources of secondary metabolites that can function as drugs or pesticides. Scientists worldwide attempted to isolate novel compounds from microorganisms; however, only less than 1% of all existing microorganisms have been successfully identified or characterized till now. Despite the limitations and gaps in knowledge, in recent years, many *Bacillus velezensis* isolates were identified to harbor a large number of biosynthetic gene clusters encoding gene products for the production of secondary metabolites. These chemically diverse bioactive metabolites could serve as a repository for novel drug discovery. More specifically, current projects on whole-genome sequencing of *B. velezensis* identified a large number of biosynthetic gene clusters that encode enzymes for the synthesis of numerous antimicrobial compounds, including lipopeptides and polyketides; nevertheless, their biological applications are yet to be identified or established. In this review, we discuss the recent research on synthesis of bioactive compounds by *B. velezensis* and related *Bacillus* species, their chemical structures, bioactive gene clusters of interest, as well as their biological applications for effective plant disease management.

## 1. Introduction

*Bacillus velezensis* was first identified in 2005 by Ruiz-García et al. by isolating two novel *Bacillus* species from environmental samples of the Vélez river [1]. This bacterium is known to exert antagonistic effects against plant pathogens via production of diverse antimicrobial compounds [2,3,4]. In 2016, several other *Bacillus* species previously classified as *B. amyloliquefaciens* subsp. *plantarum*, *B. methylotrophicus*, and *B. oryzicola* were re-classified as strains of *B. velezensis* [5]. Phylogenetic analysis based on RNA polymerase beta-subunit gene sequence and core genome, revealed that *B. velezensis* belongs to a conspecific group consisting of *B. velezensis*, *B. methylotrophicus*, and *B. amyloliquefaciens* subsp. *plantarum* FZB42 (reclassified as *B. velezensis* FZB42); however, it is distinct from the closely related species of *B. subtilis*, *B. amyloliquefaciens*, and *B. siamensis* [6]. 

**Figure 1:**
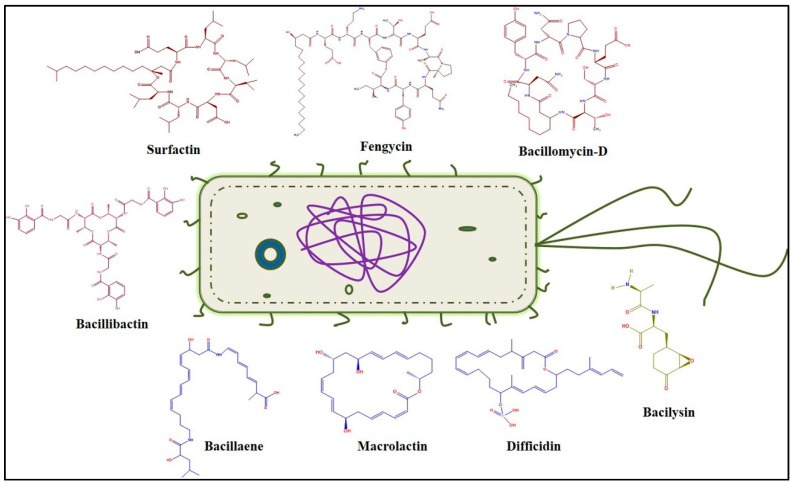
Antimicrobial compounds synthesized by *B. velezensis*. The compounds highlighted in red are synthesized by non-ribosomal peptide synthetases (NRPSs); blue compounds are synthesized by polyketide synthase (PKSs); green color compound bacilysin is synthesized by a ribosome independent pathway.

The plant-associated *B. velezensis* FZB42 genome was first sequenced in 2007, which revealed the presence of nine giant gene clusters representing approximately 10% of the whole genome. These biosynthetic gene clusters (i.e., *srf*, *bmy*, *fen*, *dhb*, *bac*, *mln*, *bae*, *dfn*, and *nrs*) encode the biosynthetic enzymes for the antimicrobial compounds, namely surfactin, bacillomycin-D, fengycins, bacillibactin, bacilysin, macrolactin, bacillaene, difficidin, and a putative peptide with unknown functions, respectively (Figure 1) [7]. Among the nine gene clusters, five encode the biosynthetic enzymes that are involved in the synthesis of non-ribosomal lipopeptides (LPs), where synthesis takes place on large enzyme complexes of non-ribosomal peptide synthetases (NRPSs). LPs share similar structures consisting of a hydrophilic peptide portion linked to the hydrophobic fatty acid chain, which could be divided into three major sub-families based on the amino acid sequence—surfactins (*srf*), bacillomycin-D (*bmy*), and fengycins (*fen*) or plipastatins (*pps*) [8]. Moreover, three more polyketide synthase (PKSs) gene clusters were identified that directed the synthesis of polyketides (PKs), e.g., macrolactin (*mln*), bacillaene (*bae*), and difficidin (*dfn*) [9,10]. PKSs and NRPSs function as multi-enzyme complexes that sequentially combine malonyl derivatives and amino acids, respectively. These tailoring enzymes employ different building blocks to synthesize a variety of secondary metabolites with therapeutic potential [11]. The products of *bac* gene cluster guide the synthesis and export of the antibacterial dipeptide bacilysin [10]. In *B. velezensis*, all of the three LP and three PK type compounds are biosynthesized via the 4’-phosphopantetheine transferase (Sfp) pathway [12]; however, the production of antibacterial compound bacilysin is independent of this pathway [13]. In addition, two other ribosomally-synthesized bacteriocins classified as amylocyclicin and plantazolicin were identified in *B. velezensis*, displaying high antibacterial activity against closely related gram-positive bacteria [13,14].

Genome mining of *B. velezensis* LM2303 revealed 13 biosynthetic gene clusters encoding the enzymes for the production of the secondary metabolites, with biocontrol potentiality against the pathogenic fungus *Fusarium graminearum* [3]. The production of the metabolites was further confirmed by chemical analysis using ultra-high-performance liquid chromatography-electrospray ionization (ESI)-mass spectrometry (MS) [3]. Among them, three gene clusters encode the enzymes for antifungal metabolites (i.e., surfactin A, iturin A, and fengycin B); eight gene clusters encode the enzymes for antibacterial metabolites (i.e., difficidin, bacilysin, bacillaene, macrolactin, plantazolicin, kijanimicin, butirosin, and surfactin A); and another three gene clusters encode the enzymes for the synthesis of metabolites involved in nutrient uptake (i.e., bacillibactin, teichuronic acid, and molybdenum cofactor) [3]. Under field conditions, LM2303 exhibited strong biocontrol efficacy against *F. graminearum*, by greatly reducing the incidence of *Fusarium* head blight, with a control efficiency of approximately 72.3% [3].

Apart from the specific antagonistic activity of *B. velezensis* against pathogenic microbes (Table 1), this bacterium was also found to contribute to plant protection by competing with harmful microorganisms for vital nutrients like iron, through the secretion of the siderophore bacillibactin (*dhb*) [15]. Endophytic *B. velezensis* CCO9 is widely distributed in various parts of the plant body, including cortex, xylem vessel, stems, and leaves, and is known for its protective functions against wheat plant diseases. It was reported that the strain CCO9 stimulates plant resistance and shows 21.64% and 66.67% disease-control efficacy of spot blotch and take-all, respectively [16]. *B. velezensis* can express induced systemic resistance (ISR) in plants by activating the defense-associated genes of jasmonic acid (JA) and salicylic acid (SA) [17]. *B. velezensis* PEA1 demonstrated both the antifungal and antiviral activities against *Fusarium oxysporum* and cucumber mosaic virus (CMV) MN594112 (capable to infect ~1200 plant species around the world), respectively. PEA1 was able to reduce the accumulation of viral coat protein (i.e., CMV-CP) by 2.1 fold, compared to untreated *Datura stramonium* plant leaves, and it also induces ISR [18]. Most notably, strains of *B. velezensis* possess genes encoding the enzymes for the production of bioactive compounds related to biocontrol traits acting in the rhizosphere. These genes are activated by exposure to root exudates, following pathogen attacks through the regulation of specific genes, rather than the presence or absence of specific genes [19].

*B. velezensis* FZB42 is distinguished from the model *B. subtilis* 168 strain by the ability to suppress the competitive organisms present in the rhizosphere, and helps in plants growth promotion [9]. Despite the high genomic similarity between *B. velezensis* and *B. subtilis*, non-plant associated *B. subtilis* species contribute only 4–5% of genome ability to the synthesis of antimicrobial compounds; however, *B. velezensis* devotes 10% of its genome to the synthesis of antimicrobial molecules [38]. In recent years, based on phylogenomic analysis of *Bacillus* genomes, many *B. subtilis* strains (e.g., *B. subtilis* 83, *B. subtilis* BZR 517 etc.) were re-classified as plant-associated *B. velezensis* species [39,40]. Moreover, several *B. subtilis*-based commercial biocontrol agents like Serenade^®^ (*B. subtilis* QST713), Kodiak™ (*B. subtilis* GB03), Taegro^®^ (*B. subtilis* var. *amyloliquefaciens* FZB24) were re-categorized as *B. velezensis*-based biocontrol agents for agricultural applications (Table 2). These commercial biocontrol agents are widely used to control various pathogenic microorganisms in soil and to protect plants from various foliar bacterial and fungal diseases, during agricultural applications.

In this review, the biosynthesis of antimicrobial compounds from *B. velezensis* and their antimicrobial activities are described. The antimicrobial compounds can be utilized as biocontrol agents for several agricultural purposes, to eradicate pathogenic microbes. More specifically, we will discuss the past and recent developments in the biosynthesis of LP- and PK-type compounds from *B. velezensis* and their biological applications, by studying the modes of actions, based on previously published reports.

## 2. Antimicrobial LPs Synthesized by *B. velezensis*

LPs produced by *B. velezensis* are categorized into three distinct families based on the amino acid sequence: surfactins, fengycins, and bacillomycin-D that were originally isolated from *B. subtilis* [49]. Many microbial LPs are assembled by ribosome-independent pathways through a series of giant enzyme machines known as NRPSs that comprise ~1000 amino acids [49]. NRPSs catalyzes the stimulation of specific amino acids by conversion into corresponding aminoacyl thioesters and the subsequent formation of peptide bonds between activated amino acids [50]. NRPSs are a multi-functional enzyme complex with at least four critical domains essential to direct the non-ribosomal synthesis of peptides. The adenylation (A) domain is the first catalytic domain that activates specific amino acids; the thiolation (T) domain is needed for amino acid tethering; the condensation (C) domain assists in peptide bond formation; and finally, the thioesterase domain (TE) contributes in chain elongation and release of the cyclic peptide [51,52]. 

### 2.1. Surfactins

The history of surfactin dates back to 1968, when it was first purified and characterized by Arima et al., as a new bioactive compound in the culture broth of *B. subtilis* [53]. To date, several surfactin-producing strains are reported from different *Bacillus* spp., including *B. velezensis*, *B. amyloliquefaciens*, *B. licheniformis*, *B. methylotrophicus*, and *B. thuringiensis* [54]. These amphiphilic cyclic LPs comprise a hydrophilic heptapeptide ring structure consisting of the amino acid sequence (Glu-Leu-Leu-Val-Asp-Leu-Leu) attached to a β-hydroxy fatty acid moiety, usually between C-13 and C-16 [55]. There are three distinct forms of surfactins (e.g., surfactin A, B, and C) that are classified, based on variations in the amino acid sequence. The amino acids, namely L-leucine, L-valine, and L-iso-leucine are present in surfactin A, B, and C, respectively, at the position of the amino acid involved in formation of the lactone ring [56]. Surfactins are synthesized by a complex interaction of NRPSs encoded by *srfA* operon, consisting of four open reading frames (ORFs), namely *srfAA*, *srfAB*, *srfAC*, and *srfAD* [57]. Among them, *srfAA*, *srfAB*, and *srfAC* ORFs encode the modular enzymes responsible for integrating the seven amino acids into the peptide ring. However, the terminal ORF *srfAD*, a repair enzyme, encodes a thioesterase/acyltransferase domain that regulates the initiation of surfactin biosynthesis [58].

**Figure 2:**
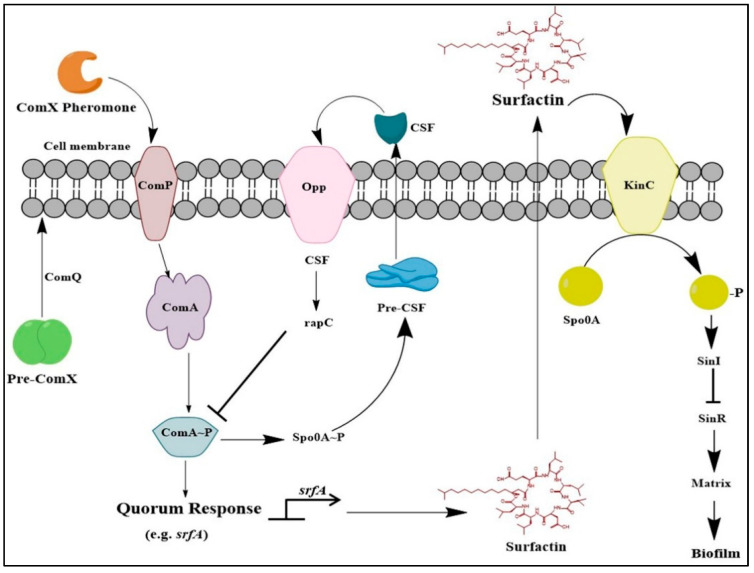
General pathway that regulates the transcription of *srfA* operon, which involves extracellular peptide regulated quorum sensing in *B. velezensis* and *B. subtilis*. T-bars show the negative regulation of protein interactions; the bent arrow indicates the function of the promoter.

Isolates of *Bacillus* spp. produce small amounts of surfactin (<10% of its biomass) that serve as a signaling molecule during inter- or intra-species interactions [59]. Surfactin biosynthesis depends on cell density; however, quorum sensing (QS) [60] prevents the constant production of bacterial cells, thereby, limiting the overall yield of surfactin (Figure 2) [59]. 

In general, *Bacillus* cells secrete extracellular signaling factors like ComX pheromones (10-amino-acid modified peptides) continuously into the liquid media. A membrane-anchored histidine kinase receptor, ComP, detects the ComX at a vital concentration and subsequently autophosphorylates its cognate receptor regulator ComA. ComA is a part of the signaling cascade system of ComQXPA that is responsible for QS in several *Bacillus* spp. Successively, phosphorylated ComA (ComA~P) triggers the transcription of the *srfA* operon by binding to the promoter site, and initiates surfactin biosynthesis [57]. However, surfactin indirectly interacts with sensor kinase KinC, followed by the phosphorylation of the master response regulator Spo0A. Phosphorylated Spo0A, subsequently, induces the expression of SinI, which antagonizes the repressor SinR that causes the transcription of genes involved in matrix biosynthesis [61]. Thus, surfactin act as a paracrine signaling molecule that triggers other cells to produce the extracellular matrix and inhibit the biosynthesis of surfactins [62]. Paracrine signaling is observed in some bacterial populations, in which ComX indirectly induces the production of extracellular matrix, in a sub-population of cells, but these surfactant-responsive cells can no longer respond to ComX, thus, halting the production of additional surfactin [62]. 

In addition to the ComX-dependent regulation, several other factors including competence and sporulation-stimulating factor (CSF) and aspartate phosphatase (Rap) proteins, including Rap C, D, F, and H, also regulate the surfactin biosynthesis. CSF is a species-specific extracellular peptide secreted by *Bacillus* spp. and imported into the cell by oligopeptide permease (Opp; also known as Spo0K) [63]. Subsequently, CSF binds to the Rap proteins, which dephosphorylates ComA~P, thereby, impairing its function. However, the dephosphorylation of ComA~P can be inhibited to promote the transcription of *srfA* gene and surfactin biosynthesis [64]. These mechanisms would rationally explain why most *Bacillus* spp. in the liquid culture medium show minimal surfactin biosynthesis (Figure 2).

**Figure 3:**
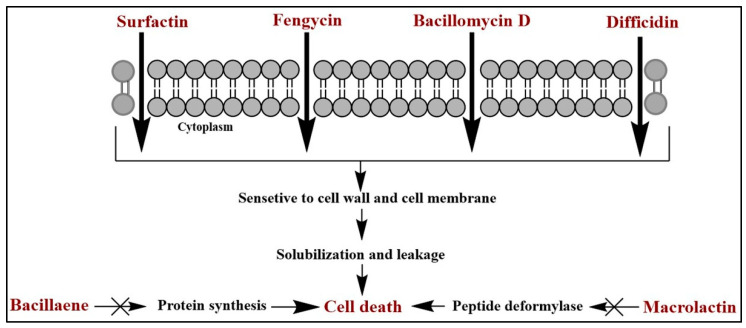
Antimicrobial mechanisms of lipopeptides (LPs) and polyketides (PKs) synthesized by *B. velezensis.*

As a consequence of its amphiphilic structure, surfactin is a powerful and effective bio-surfactant molecule displaying antimicrobial activity against a wide variety of pathogenic microbes (Figure 3), including *Ralstonia solanacearum* [20], *Pseudomonas syringae* pv. *tomato* DC3000 [23], and *F. verticillioides* [22]. Additionally, surfactin was shown to harbor anti-mycoplasma activity against *Mycoplasma hyorhinis* [65], and anti-*Legionella* activity against *Legionella pneumophila* [66]. In a similar study, surfactins (surfactin B and C) produced by *B. velezensis* 9D-6, inhibited the growth of *P. syringae* DC3000 and *Clavibacter michiganensis*, during an in vitro plate assay. Furthermore, co-cultivation of *B. velezensis* 9D-6 and *P. syringae* DC3000, substantially reduced root colonization of DC3000 in *A. thaliana* seedlings, signifying that 9D-6 employs additional non-antimicrobial mechanisms against phytopathogens [21]. Upon root colonization, the strain *B. subtilis* 6051 protects Arabidopsis plants from pathogenic bacteria *P. syringae* DC3000 infection, and reduces plant mortality by 70%, through the combined actions of biofilm formation and surfactin secretion. The level of LPs secreted by *B. subtilis* 6051 was sufficient to kill the pathogen [23].

### 2.2. Fengycins

Fengycin or plipastatin, originally discovered from *B. subtilis* F-29-3 in 1986 is known to exhibit antifungal activity against a broad spectrum of filamentous fungi [67]. The structure of fengycins is composed of cyclic octapeptide containing decapeptides linked to N-terminal β-hydroxy fatty acid chain, usually between C-12 and C-19 [68]. Two isoforms of fengycin, fengycin A and fengycin B differ structurally, due to the presence of Ala/Val dimorphy at the sixth position [69]. Fengycins are synthesized by NRPSs encoded by an operon consisting of five ORFs *fenA-E* or *ppsA-E* [49].

Fengycins are assumed to cause cell death of the target organism by interacting with the cell membrane and altering the cell permeability. The findings of scanning electron microscopy (SEM) and transmission electron microscopy (TEM) suggested that treatment of hyphal cells of *Magnaporthe grisea* with fengycin (20 µg/mL) from *B. subtilis* BS155, led to the ultrastructural destruction of pathogen hyphae and the loss of cytoplasm, plasma membrane, or cell membrane integrity, which eventually resulted in cell lysis [8].

The antibiotic LP fengycin can be used to treat various plant diseases, e.g., barley head blight disease (*F. graminearum*) [70], rice blast disease (*Magnaporthe grisea*) [8], gray mold disease (*Botrytis cinerea*) [24], maize disease (*Rhizomucor variabilis*) [25], and cucurbit powdery disease (*Podosphaera fusca*) [71], etc. Fengycins produced by *B. velezensis* SQR9, exhibited antagonistic activities against *F. oxysporum*, *F. solani*, and *Phytophthora parasitica* and *Verticillium dahliae* Kleb [15] Plipastatin A synthesized by *B. amyloliquefaciens* S76-3 demonstrated superior fungicidal activity against *F. graminearum*, by inactivating the conidial spores at a minimum inhibitory concentration of 100 μg/mL. Microscopy experiments showed marked morphological changes in conidia and major distortions in the *F. graminearum* hyphae, with increased vacuolation [72]. However, in contrast to the antifungal activity of this LP, the antibacterial activity of fengycins produced by *B. amyloliquefaciens* MEP_2_18 against the spot disease-causing *Xanthomonas axonopodis* pv. *vesicatoria* in tomato plants were characterized using liquid chromatography ESI-MS/MS [26].

### 2.3. Bacillomycin-D

Bacillomycin-D belongs to the LPs iturin family, including iturin A, C, D, and E, bacillomycin-F and L, bacillopeptin, and mycosubtilin [73]. This antimicrobial compound is a cyclic heptapeptide bound to the β-amino fatty acid chain between C-15 and C-18. The *bmy* operon that regulates the biosynthesis of bacillomycin-D comprises four genes (i.e., *bmyD*, *bmyA*, *bmyB*, and *bmyC*) without orthologues in *B. subtilis* 168 [74]. Most notably, the *bmy* gene cluster encoding the enzymes for the synthesis of bacillomycin-D, is separated from fengycin gene cluster, by only 25 kb, within the *B. velezensis* FZB42 genome, and is positioned exactly at the same location of the iturin-A gene cluster of *B. subtilis* RB14 [73]. Three pleiotropic regulators (i.e., DegU, DegQ, and ComA) and two sigma factors (i.e., σB and σH) positively regulate the transcriptional activation of the *bmy* promoter towards the synthesis of bacillomycin-D. Another study demonstrated the role of DegU and ComA in regulating the bacillomycin-D expression. Inactivation of the genes encoding DegU and ComA proteins resulted in an impaired promoter function of the *bmy* operon. As a consequence, the transcription rate of the *bmy* operon was three to four fold lower in the mutant derivatives than in parental *B. velezensis* FZB42 strain [74]. Furthermore, LP bacillomycin-D synthesized by *B. velezensis* SQR9 acts as a signaling molecule in biofilm formation, due to an increase in the intracellular iron concentration and activation of the KinB-Spo0A-SinI-SinR signal cascade-based synthesis of biofilm matrix components [75]. 

Bacillomycin-D synthesized by *B. velezensis* were shown to display antimicrobial activity against different microorganisms, such as *X. campestris* pv. *cucurbitae* [29], *Aspergillus flavus* [76], *F. graminearum* (Fusarium head blight) [12], *F. oxysporum* f. sp. *cucumerinum* (vascular wilt in cucumber plants), etc. [30]. Mutant strains deficient in the production of bacillomycin-D compromised antifungal action, suggesting the role of bacillomycin-D in the antifungal activity of FZB42. SEM and TEM analyses confirmed that bacillomycin-D causes morphological alterations in the cytoplasmic membranes and cell walls of *F. graminearum* hyphae and conidia. This resulted in the accumulation of reactive oxygen species (ROS), and ultimately triggered the cell lysis of *F. graminearum*. The 50% effective concentration (EC_50_) that purified bacillomycin-D and inhibited the activity of *F. graminearum* was estimated to be about 30 μg/mL [12].

## 3. Antibacterial PKs Synthesized by *B. velezensis*

PKs are a natural class of secondary metabolites synthesized by PKSs. To date, more than 10 thousand PK-type compounds are identified from bacteria, fungi, plants, and animals, of which at least 20 were developed as commercial drugs including erythromycin, tetracycline, and lovastatin [77]. The genes encoding PKSs were identified in 1993 during genome sequencing of *B. subtilis* 168. PKSs catalyzes the decarboxylative Claisen condensation reactions with possible additional alterations through β-reduction, dehydration, or enoyl-reduction reactions that are catalyzed by some PKSs-modifying domains. The multi-enzyme system of PKSs uses acyl carrier proteins that are post-translationally modified with the 4’-phosphopantetheine prosthetic group, to guide the intermediate PK molecule throughout the elongation process [9]. Interestingly, the model strain *B. subtilis* 168 was shown to contain a large PKS gene cluster designated as *pksX*; however, this strain was not capable of synthesizing PKs due to mutation in the *sfp* gene encoding 4’-phosphopantetheine transferase (Sfp) [9].

### 3.1. Bacillaene

Bacillaene, a novel polyene antibiotic, was discovered from the fermentation broth of *B. subtilis* that inhibit the prokaryotic protein synthesis, by an unknown mechanism [78]. Among the three giant modular PKSs system in *B. velezensis* (*pks1*, *pks2*, *pks3*), bacillaene is synthesized by the enzymes encoded by the *pks1* (*bae*) gene cluster, which is an ortholog of the *pksX* gene cluster of *B. subtilis* 168 [9]. Despite antibacterial activity of this antibiotic against multi-drug-resistant bacterial isolates, for many years, characterization of bacillaene using the traditional methods based on fractionations was proved challenging, owing to its chemical instability [11]. On exposure to light or room temperature, bacillaene decomposes rapidly, which hindered earlier attempts to identify the biosynthetic pathway of this antibiotic molecule [79]. Antibacterial polyketide bacillaene synthesized by *B. velezensis* FZB42, exhibited a minor extent of bacteriostatic effect against *Erwinia amylovora*, a causal agent of fire blight disease [32]. In addition, bacillaene-A synthesized by *Bacillus* spp. displayed antifungal activity against *Termitomyces* fungi [33].

### 3.2. Macrolactin

Macrolactin was originally isolated from the ethyl acetate extract of an unclassified deep-sea bacterium *Bacillus* spp. Sc026 [80]. In *B. velezensis*, the *pks2* (*mlnBCDEFGH*) gene cluster encode the enzymes for antibacterial compound macrolactin, which is an inhibitor of the bacterial peptide deformylase [7,81]. The chemical structure of macrolactin is synthesized by the expansion of the acetyl starter unit, by 11 successive Claisen condensation reactions with malonyl-CoA. Currently, approximately 17 different types of macrolactins are identified; however, only four macrolactin forms (e.g., macrolactin-A, macrolactin-D, 7-O-malonyl-macrolactin-A, and 7-O-succinyl-macrolactin) are found in *B. velezensis*. Of the four, 7-O-malonyl-macrolactin-A was found to have bacteriostatic effects on a variety of gram-positive and multidrug-resistant bacterial pathogens, particularly, methicillin-resistant *Staphylococcus aureus*, vancomycin-resistant enterococci, and small-colony variant of *Burkholderia cepacia* [34].

### 3.3. Difficidin

Difficidin was detected for the first time in the fermentation broth of *B. subtilis* ATCC-39320 and categorized as an unsaturated macrocyclic polyene lactone phosphate ester in its 22-member family [82]. Difficidin, as well as its oxidized form oxydifficidin, encoded by the enzymes of *pks3* (*dif*) gene cluster, appeared primarily as their alkali ion adducts in the matrix-assisted laser desorption ionization-time of flight mass spectra [9]. Oxydifficidin has a hydroxyl group at the fifth position of the difficidin ring structure [9].

The antibiotic compounds, difficidin and bacilysin, exhibited antibacterial activity against two rice pathogens, *X. oryzae* pv. *oryzae*, as well as *X. oryzae* pv. *oryzicola*, causing bacterial blight and bacterial leaf streak disease, respectively. In combination, these two compounds affected the cell wall of *Xanthomonas*, as indicated by SEM and TEM observations. Furthermore, the quantitative real-time PCR results also indicated the downregulation of several *X. oryzae* genes including *rpfF*, *gumD*, *glmS*, *ftsZ,* and *rrlA*, related to the virulence, cell division, and biosynthesis of proteins and cell wall of *X. oryzae* [35]. In a similar study, a butanolic extract of the *B. velezensis* DR-08 broth culture containing difficidin and oxydifficidin displayed antibacterial activity against *R. solanacearum*, a leading causal agent of tomato bacterial wilt with a minimum inhibitory concentration (MIC) value of 12.62 μg/mL. Furthermore, the metabolic extract of this bacterium also inhibited the growth of 14 phytopathogenic bacteria with MIC values ranging from 1.95–500 μg/mL [36].

## 4. Bacillibactin

Iron is an essential element for all living organisms and serves as a vital cofactor to perform cellular processes including DNA synthesis, respiration, and defense against ROS [83]. Several *Bacillus* spp. secretes bacillibactin, the catecholic iron siderophore, which is very important in facilitating Fe(III) acquisition, especially when the *Bacillus* cells experience iron limitation [84]. In *B. velezensis*, the products of the functional *dhb* gene cluster was shown to assist in the synthesis of bacillibactin (small molecule iron-chelators). It is a part of a complex transport system that enables the *B. velezensis* cells to accumulate iron ions and acquire them from their natural environment, under iron-limiting conditions [10]. LPs (i.e., bacillomycin D, fengycins, and surfactins) coupled with bacillibactin synthesized by *B. velezensis* SQR9 had an antagonistic effect against certain fungal pathogens, including *F. oxysporum*, *F. solani*, *P. parasitica*, where the production of bacillibactin was greatly upregulated. However, mutant strains deficient in LPs and bacillibactin displayed a substantial reduction in antifungal effects, when challenged with these fungal pathogens. These results suggest that bacillibactin plays a passive role in the suppression of microbial pathogens, either by depriving them of essential iron or directly inhibiting the growth [15]. However, there is no experimental evidence of antimicrobial activity of purified bacillibactin, in the absence of known secondary metabolites like LPs or PKs.

## 5. Bacilysin

Bacilysin is a Trojan horse antibiotic, synthesized by the enzymes of the *bacA-E* gene cluster (formerly *ywfBCDEF*) of certain *Bacillus* spp. [85]. This dipeptide antibiotic [L-alanyl-(2,3-epoxycyclohexanone-4)-L-alanine] was first isolated from the soil bacterium *B. subtilis* by Foster and Woodruff in 1946 [35], and its structure was established by Walker and Abraham in 1970 [86]. 

**Figure 4:**
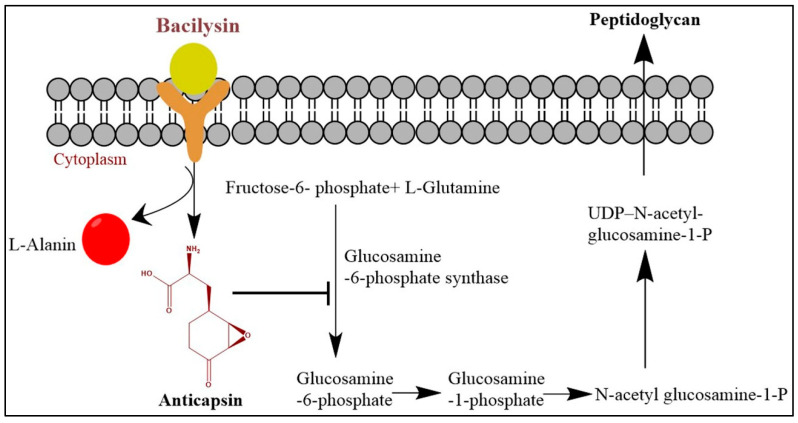
Modes of actions of antibacterial activity of bacilysin synthesized by *B. velezensis*.

Bacilysin relies on peptide transporters for uptake into the target cells. Once internalized into the susceptible cells, bacilysin is hydrolyzed by cytoplasmic peptidases to non-proteinogenic anticapsin (epoxy-cyclohexanonyl-Ala) and N-terminal L-alanine (Figure 4). The C-terminal epoxy amino acid (anticapsin) of bacilysin is responsible for the antimicrobial activity against pathogenic microorganisms [86]. Anticapsin covalently interacts with the active site of the cell wall biosynthetic enzyme glucosamine synthase, the latter catalyzes the synthesis of glucosamine-6-phosphate from fructose-6-phosphate and glutamine [87]. This covalent binding was caused by the crosslinking between the active thiol of cysteine residue present in the enzyme glucosamine synthase, and the apoxide functional group of anticapsin. Therefore, the bacterial peptidoglycan or fungal mannoprotein biosynthetic pathway was thus blocked, leading to cell protoplasting and lysis [88]. In real-time PCR analysis, it was confirmed that several genes, including *glmS*, *psbA1*, *mcyB*, and *ftsZ*, which are related to the biosynthesis of peptidoglycan, cell division, and photosynthesis in *Microcystis aeruginosa* cells, were downregulated in response to bacilysin treatment (4 mg/L) [88].

The antimicrobial action of bacilysin depends on the composition of culture medium and the activity could be reversed by using some antagonists like N-acetylglucosamine, several dipeptides, and amino acids, which might inhibit the transport of this antibiotic into the microbial cells [89]. Bacilysin, synthesized by *B. velezensis* FZB42, exerted antagonistic activities against *S. aureus* and *C. michiganense* subsp. *Sepedonicum*, which cause ring rot disease in potatoes [37]. In a similar study, bacilysin synthesized by *B. velezensis*, exhibited strong anti-cyanobacterial activity against *M. aeruginosa*, which cause harmful algal blooms with a kill rate of 98.78%. However, disruption of a single gene *bacB* or supplementation of N-acetylglucosamine to the bioassay plates, abolished the inhibitory effect of bacilysin [88]. Analyses using SEM and TEM revealed that exposing *X. oryzae* pv. *oryzae* and *X. oryzae* pv. *oryzicola* to 50 μg/mL of bacilysin for 12 h, triggered changes in the cell wall structure as well as efflux of intracellular components [35]. In a similar study, TEM revealed the micro- and ultra-structural changes to *M. aeruginosa* cells treated with 15 mg/L bacilysin for 2 h. The cells were severely damaged and the cytoplasm was condensed, eventually, resulting in plasmolysis of *M. aeruginosa* cells [88].

## 6. Conclusions

Over the past few decades, hundreds of antimicrobial drugs were developed from a plethora of microorganisms. These antimicrobial molecules are considered safe for the treatment of various plant diseases, due to their broad-spectrum activity against multiple microbes, reduced toxicity compared to chemical pesticides, environment-friendly nature, and reduced risk of resistance acquisition in pathogenic microbes. Recently characterized bioactive compounds synthesized by *B. velezensis* demonstrated promising antimicrobial activities suitable for agricultural applications; therefore, the mode of actions of these antimicrobial compounds against various plant pathogens were extensively investigated. Although, some *B. subtilis* strains are also capable to produce bioactive secondary metabolites, it was reported that the biosynthetic arsenals of *B. velezensis* is more powerful and diverse than that of *B. subtilis*. In addition, in recent years, several biocontrol agents that were formulated from *B*. *subtilis* strains, were reclassified as *B. velezensis*-dependent biocontrol agents, based on the availability of genome sequence data. Taken together, *B. velezensis* could be a versatile and powerful biocontrol agent that can be used as an effective alternative to synthetic agrochemicals, either by using the bacteria itself or by extracting its active compounds. Moreover, the elucidation of the genes responsible for the synthesis of bioactive compounds and strategies to alter these genes using genome-engineering techniques would constitute additional important measures to increase the biosynthesis of metabolites in *B. velezensis*.

## Figures and Tables

**Table 1:** Antimicrobial molecules synthesized by *B*. *velezensis* to control pathogenic microbes.

Compounds	Gene Clusters	Antimicrobial Activity				References
		Antibacterial Activity (Diseases)	MIC (Pathogens)	Antifungal Activity (Diseases)	MIC (Pathogens)	
Surfactins	*SrfA-D*	*Cochliobolus carbonum* (Leaf spot); *P. syringae* pv. *tomato*; *R. solanacearum*	25-100 µg/mL (*P. syringae* pv. *tomato*)	*F. verticillioides* (Maiz disease)	-	[20,21,22,23]
Fengycins	*FenA-E*	*R. solanacearum* (Tomato wilt), *X. euvesicatoria* (Pepper spot); *X. axonopodis* pv. *esicatoria*	-	*F. oxysporum* (banana Fusarium wilt); *F. graminearum* (Fusarium head blight); *B. cinerea* (Grey mould); *R. variabilis* (Maiz disease)	20.0 µg/mL (*M. grisea*); 100 µg/mL (*F. graminearum*)	[2,24,25,26,27,28]
Bacillomycin-D	*bmyA-D*	*X. campestris* pv. *cucurbitae*	-	*Colletotrichum gloeosporioides* (Bitter rot); *F. graminearum* (Fusarium head blight); *F. oxysporum* f. sp. *cucumerinum* (Cucumber vascular wilt)	30 μg/mL (*F. graminearum*)	[12,29,30,31]
Bacillaene	*baeBCDE*, *acpK*, *baeGHIJLMNRS*	*E. amylovora* (Fire blight)	-	*Termitomyces* spp.	-	[32,33]
Macrolactin	*MlnA-I*	*S. aureus*; *B. cepacia*	-	-	-	[34]
Difficidin	*dfnAYXBCDEFGHIJKLM*	*X. oryzae* pv. *oryzae* (Rice blight) and *X. oryzae* pv. *oryzicola* (Rice leaf streak); *E. amylovora* (Fire blight); *R. solanacearum* (Tomato wilt)	12.62 μg/mL (*R. solanacearum*);	-	-	[32,35,36]
Bacilysin	*bacA-E*	*S. aureus* and *C. michiganense*; *X. oryzae* pv. *oryzae* (Rice blight) and *X. oryzae* pv. *oryzicola* (Rice leaf streak); *E. amylovora* (Fire blight)	50.0 μg/mL (*X. oryzae* pv. *oryzae* and *X. oryzae* pv. *oryzicola*)	-	-	[32,35,37]

**Table 2:** Commercial uses of *B. velezensis*-based biological control in agriculture.

Commercial Name	Biocontrol Agents	Current Name of Biocontrol Agents (*NCBI Accession Number)	Target Pathogens (Disease)	Manufacturer	References
RhizoVital^®^	*B. amyloliquefaciens* FZB42^T^	*B. velezensis* FZB42^T^ (CP000560.2)	*R. solani* (Bottom rot in lettuce); *E. amylovora* (Fire blight disease)	ABiTEP, GmbH, Berlin, Germany	[20,41]
Botrybel	*B. velezensis*	*B. velezensis*	*B. cinerea* (Gray mold)	Agricaldes, Spain	[42]
Serenade^®^	*B. subtilis* QST713	*B. velezensis* QST713 (CP025079.1)	*Trichoderma aggressivum*; *Blumeria graminis* (Powdery mildew)	AgraQuest Inc., California, USA	[43,44]
Kodiak™	*B. subtilis* GB03	*B. velezensis* GB03 (AYTJ00000000)	*F. oxysporum* (Fusarium-wilt); *R. solani* (Cotton disease)	Gustafson Inc., Texas, USA	[45]
Taegro^®^	*B. subtilis* var. *amyloliquefaciens* FZB24	*B. velezensis* FZB24	*F. oxysporum* (Tomato wilt); *Phytophthora infestans* (Potato late blight)	Novozymes, Virginia, USA	[46,47,48]

*NCBI: National Center for Biotechnology Information.
